# *Hif1a* and *Hif2a* can be safely inactivated in cone photoreceptors

**DOI:** 10.1038/s41598-019-52508-8

**Published:** 2019-11-06

**Authors:** Marijana Samardzija, Maya Barben, Vyara Todorova, Katrin Klee, Federica Storti, Christian Grimm

**Affiliations:** 1 Lab for Retinal Cell Biology, Department of Ophthalmology, University of Zurich, Schlieren, CH-8952 Switzerland; 2 Center for Integrative Human Physiology (ZIHP), University of Zurich, Zurich, CH-8006 Switzerland; 3 Neuroscience Center Zurich, University of Zurich, Zurich, CH-8006 Switzerland

**Keywords:** Neuroscience, Retina

## Abstract

Impaired tissue oxygenation results in hypoxia and leads to the activation of hypoxia-inducible transcription factors (HIF). A chronic, HIF-triggered molecular response to hypoxia may be an important factor in the etiology of age-related macular degeneration (AMD) and is likely activated before any clinical manifestation of the disease. Thus, HIF1 and HIF2 recently emerged as potential therapeutic targets for AMD. To address and evaluate potential consequences of anti-HIF therapies for retinal physiology and function, we generated mouse lines that have *Hif1a*, or both *Hif1a* and *Hif2a* ablated specifically in cone photoreceptors. The knockdown of *Hifs* in cones did not cause detectable pathological alterations such as loss of cone photoreceptors, retinal degeneration or abnormalities of the retinal vasculature, had no impact on retinal function and resulted in a similar tolerance to hypoxic exposure. Our data indicate that HIF transcription factors are dispensable for maintaining normal cone function and survival in retinas of adult mice. This study provides the groundwork necessary to establish safety profiles for strategies aiming at antagonizing HIF1A and HIF2A function in cone photoreceptors for the treatment of retinal degenerative diseases that involve a hypoxic component such as AMD.

## Introduction

Like other mammalian tissues, the retina requires oxygen in order to support aerobic metabolism and energy production in form of ATP. High oxygen levels are required in particular by photoreceptors for generating sufficient ATP necessary to maintain ion gradients in darkness and to execute phototransduction under light conditions. Adequate oxygen supply is especially important considering that the retina is one of the tissues with the highest oxygen consumption in the body^1,2^. While the inner retina retrieves oxygen from retinal vessels, photoreceptors in the outer retina are supplied with oxygen through the choroidal vasculature^3^. Reduced oxygenation of photoreceptors, resulting in local retinal hypoxia, has been suggested to be a contributing factor in the etiology of age-related macular degeneration (AMD)^4–6^. AMD patients lose fine acuity vision due to the loss of photoreceptors in the macula, the central retinal area that is enriched with cone photoreceptors. The exact mechanisms preceding this loss are poorly understood but tissue alterations such as reduced blood flow and dropout of vessels in the choriocapillaris^7^, retinal pigment epithelium (RPE) atrophy^8,9^, thickening as well as decreased permeability of the Bruch’s membrane^10^ and accumulation of drusen suggest that impaired oxygen supply to photoreceptors^11,12^ may be one of the crucial factors. Evidence for reduced oxygen availability and tissue hypoxia in AMD patients is, however, circumstantial and rather difficult to corroborate experimentally. While the reduced choroid blood perfusion has been clinically well documented (reviewed in^13^), the contribution of e.g. Bruch’s membrane thickening or drusen deposition to reduced tissue oxygenation, local hypoxia and the development of AMD pathology is challenging to assess. Mathematical and computational modeling may be instrumental to establish criteria and model predictions suitable for experimental validation of the contribution of hypoxic events to development and progression of AMD^14–16^. On a molecular level, such chronic hypoxic conditions likely lead to reduced mitochondrial oxygen consumption, thereby promoting glycolysis in order to maintain ATP levels. As glycolysis generates less ATP per mole glucose than oxidative phosphorylation, this may lead to local energy depletion and photoreceptor death as recently proposed^17^. In a mouse model of genetically-induced hypoxia in the RPE, Kurihara *et al*. demonstrated that a chronically activated hypoxic response in the RPE leads to gross glucose metabolism and lipid-handling defects, which not only affect the RPE but also cause metabolic perturbations in the neighboring photoreceptors^11^.

Activation of hypoxia-inducible factors (HIFs) is a hallmark of the cellular response to reduced oxygenation. HIFs are heterodimeric complex proteins consisting of an alpha subunit (HIF1A, HIF2A, or HIF3A) and a beta subunit (HIF1B, alias aryl hydrocarbon receptor nuclear translocator or ARNT), reviewed in^18^. HIFA subunits are constantly transcribed and translated but are rapidly degraded by proteasomes under normal oxygen levels. The degradation of HIFA subunits is triggered by the oxygen-dependent hydroxylation of conserved proline residues by a family of prolyl hydroxylase enzymes (PHD1, PHD2 and PHD3), reviewed in^19^). Hydroxylated HIFA subunits are recognized by von Hippel Lindau protein (VHL), which is part of an E3 ubiquitin ligase complex that polyubiquitinates the hydroxylated proteins tagging them for subsequent proteasomal degradation in normoxia. Under reduced oxygen tension PHDs are enzymatically inactive and HIFA subunits are no longer hydroxylated, i.e. not primed for degradation. Instead, HIFA proteins accumulate, translocate to the nucleus, combine with HIFB and activate expression of target genes. HIF1 in particular regulates a large variety of genes including genes that are responsible for cellular oxygen homeostasis and glucose uptake^20,21^.

We recently showed that inactivation of *Hif1a* rescues cones and rods from degenerative processes induced by activation of chronic molecular response to hypoxia caused by *Vhl* ablation^12,17^. To rescue the degenerative phenotype of a chronic hypoxic response activated in the RPE, however, *Hif2a* needed to be ablated^11^. Hence, the inhibition of HIF1 in photoreceptors and of HIF2 in the RPE has been proposed as a potential therapy to treat AMD and other hypoxia-mediated retinal degenerations^11,12^. We already showed that deletion of *Hif1a* from adult rods is safe and does not affect retinal morphology or function^22,23^. Here, we investigated the physiological consequences of HIF1A inactivation specifically in cone photoreceptors by using mouse models with either the normal rod-dominant or an all-cone retina^24^. We also tested the consequences of a simultaneous ablation of *Hif1a* and *Hif2a* in cones to further explore safety aspects of potential therapies aiming to temper the HIF-response for the treatment of AMD and other retinal diseases with a hypoxic component.

## Results

**Figure 1:**
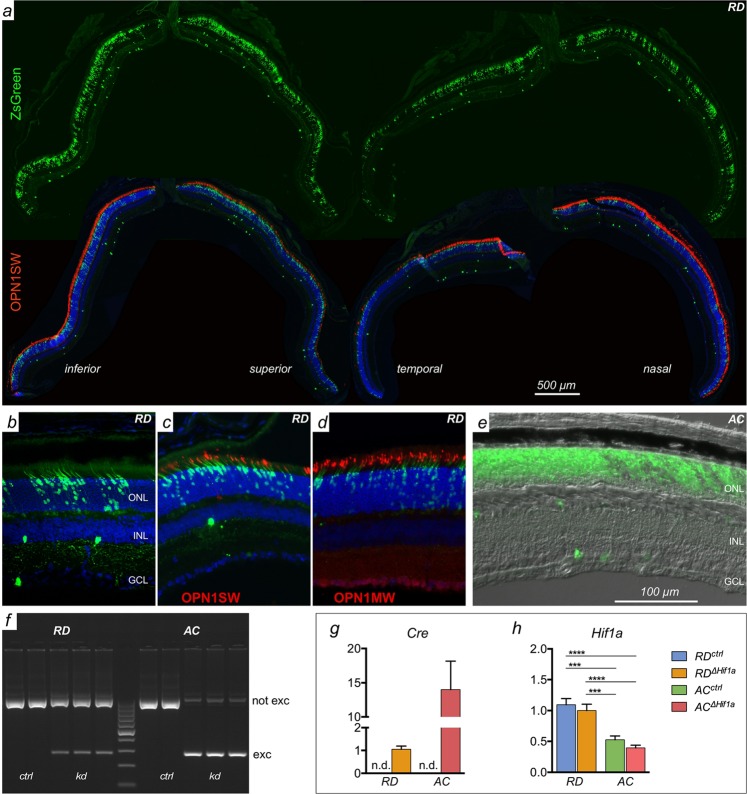
Evaluation of *Hif1a* knock-down in cones of RD^∆*Hif1a*^ and AC^*∆Hif1a*^ mice. (**a)** Immunofluorescence of retinal sections from *RD;BPCre;ZsGreen* reporter mice. Sections were cut in the dorsal/ventral (left) and temporal/nasal (right) orientation and stained for OPN1SW (red). Green fluorescence indicates cells with Cre activity. Blue: DAPI. (**b–d)** Higher resolution images of retinal sections of *RD;BPCre;ZsGreen* mice showing green fluorescence from the activated reporter alone **(b)**, or in combination with cones expressing OPN1SW **(c)** or OPN1MW **(d)**. (**e)** High resolution image of a retinal section of an *AC;BPCre;ZsGreen* mouse. (**f**) PCR amplification of *Hif1a* genomic DNA isolated from retinas of *RD*^*∆Hif1a*^, *AC*^*∆Hif1a*^ (kd) and their respective control (ctrl) mice. The floxed, not excised (not exc) *Hif1a* sequence is detected at approximately 900 bp and Cre-mediated deletion results in a fragment of 270 bp (excised, exc). Higher levels of *Hif1a* excision are expected in AC mice based on the increased number of S-cones in these mice. (**g,h)** Relative expression levels of *Cre* **(g)** and *Hif1a* **(h)** mRNA in retinas of indicated mice at 8 weeks of age. mRNA levels were normalized to *Actb* and expressed relatively to the levels in *RD*^*∆Hif1a*^ mice, which levels were set to 1. Shown are means ± SD of n = 3. One-way ANOVA and Tukey’s test for multiple comparisons was used to analyze significance. ONL: outer nuclear layer, INL: inner nuclear layer, GCL: ganglion cell layer. Scale bars: 500 µm **(a)**, and 100 µm **(b–e)**.

To address the feasibility of a therapeutic strategy targeting *Hif1a*, we analyzed the impact of cone-specific *Hif1a* inactivation on retinal morphology and function. For this purpose we deleted *Hif1a* in cone photoreceptors of mice with a normal, rod-dominant (RD) or an all-cone (AC) retina. AC mice are enriched with cone photoreceptors which should facilitate the analysis of potential effects of cone-specific *Hif1a* inactivation. Mice expressing Cre recombinase under the transcriptional control of the blue cone opsin promoter (BP^25^,) were crossed with *Hif1a*^*flox/flox*^ mice to generate *RD*^*ΔHif1a*^ and *AC*^*ΔHif1a*^ mice (see methods). To verify *Hif1a* inactivation in cones, we first evaluated CRE expression in RD and AC mice using ZsGreen reporter mice (Fig. 1a–e). CRE-activated reporter expression was strong in the ONL of both mice with occasional positive cells in the INL and GCL (Fig. 1a–e), as reported before for AC mice^12^. Immunostaining for S- and M-cone opsins (OPN1SW and OPN1MW, respectively) in the RD retina showed that the majority of cone photoreceptors was positive for ZsGreen (Fig. 1a,c,d). Indeed, a detailed analysis revealed that approximately 80% of cone photoreceptors expressed the reporter transgene (data not shown). Since no spontaneous ZsGreen expression was noticed in retinas of CRE-negative reporter mice (not shown) this indicates that CRE activity was mostly, but not exclusively confined to cones expressing S-opsin, as reported^25^.

PCR amplification of retinal genomic DNA from Cre-positive RD and AC mice with primers to detect excision of the floxed *Hif1a* sequence resulted in the production of a long fragment from not excised and a short fragment from excised *Hif1a* DNA. The respective Cre-negative controls amplified exclusively the long, not-excised fragment (Fig. 1f). This confirmed that BP-Cre recombined *Hif1a* floxed sequences and suggests predominant inactivation of *Hif1a* in S-opsin expressing cones (see above). The stronger PCR signal detected for the excised sequences in *AC*^*ΔHif1a*^ mice indicated that *Hif1a* gene inactivation occurred in more cells in AC mice, likely reflecting the higher number of CRE-expressing cones in these mice (Fig. 1e^26^,). However, this is only an indication and not a quantitative measure of excision efficiency. The 14-fold higher Cre expression in 8 week-old *AC*^*ΔHif1a*^ mice as compared to *RD*^*ΔHif1a*^ mice (Fig. 1g) supported this notion.

Interestingly, basal retinal *Hif1a* mRNA levels were significantly lower in *AC* mice than in *RD* mice (Fig. 1h, statistics not shown), suggesting that basic expression levels of *Hif1a* may be lower in cones than in rods. Even though CRE-mediated recombination inactivated *Hif1a* in cones (Fig. 1f), the reductions of *Hif1a* mRNA levels were not significant when measured in total retinal RNA, likely because other retinal cells may express *Hif1a* at higher levels concealing the photoreceptor-specific effect.

**Figure 2:**
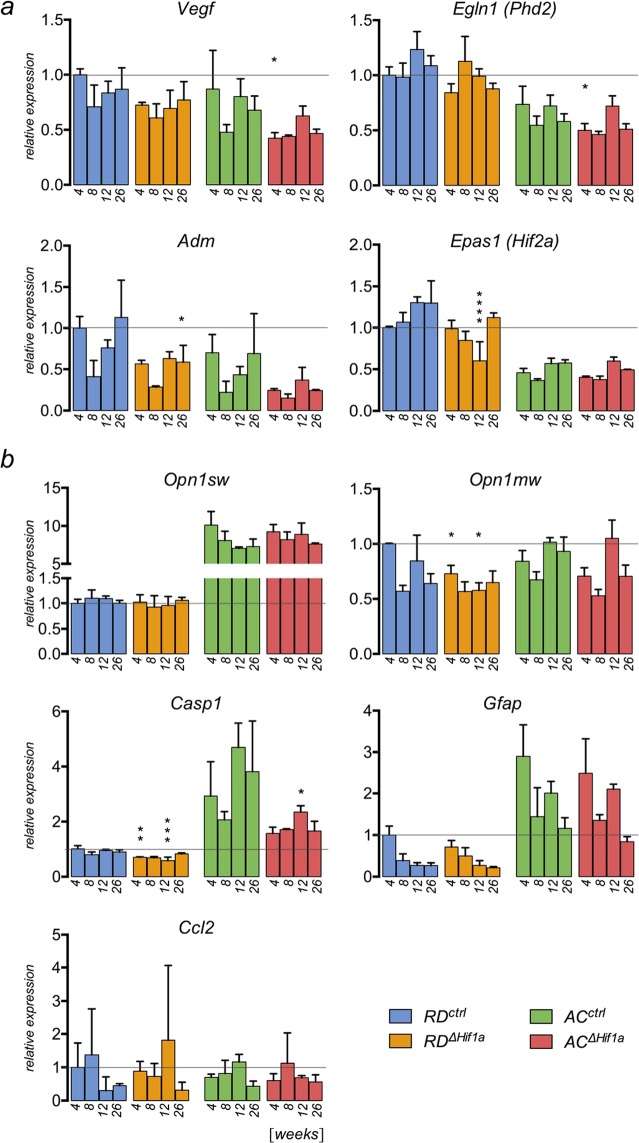
Retinal gene expression in RD^∆*Hif1a*^ and AC^*∆Hif1a*^ mice during ageing. (**a)** HIF1-responsive genes (*Vegf*, *Egln1, Adm*) and *Epas1* (*Hif2a*). (**b)** Cone-specific genes (*Opn1sw*, *Opn1mw*) and genes involved in degeneration (*Casp1*), gliosis (*Gfap*) or inflammation (*Ccl2*). Expression was determined in RD^*∆Hif1a*^ and AC^*∆Hif1a*^ and their respective controls at 4, 8, 12 and 26 weeks of age (as indicated). Expression levels were normalized to *Actb* and shown relative to 4-week-old *RD*^*ctrl*^ mice, the levels of which were set to 1 (dotted line). Shown are means ± SD of n = 3. Significance between knockdown and control mice was calculated at every individual time point by two-way ANOVA with Šídák’s multiple comparison test *p < 0.05; **p < 0.01; ***p < 0.001.

**Figure 3:**
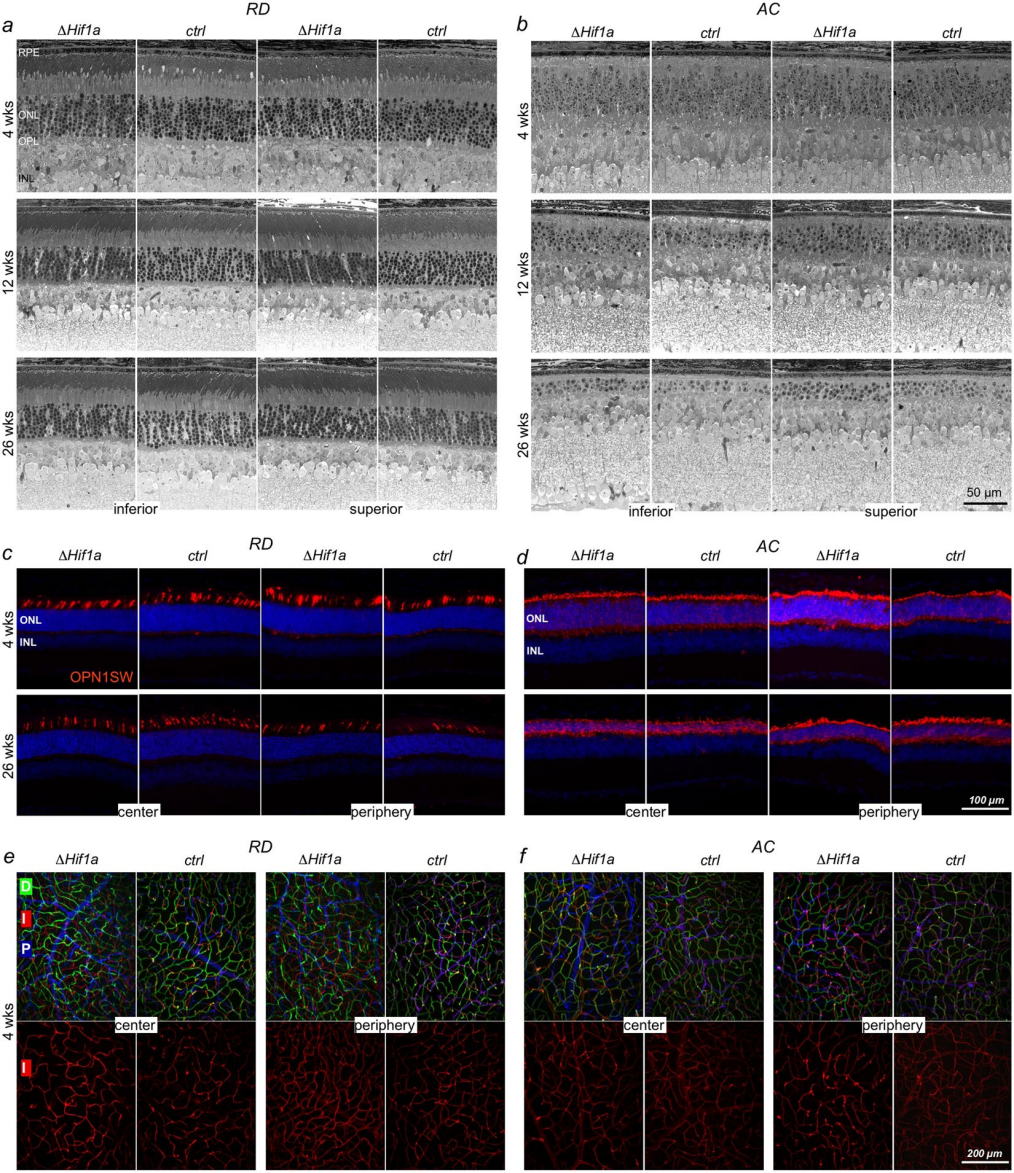
Retinal morphology following *Hif1a* inactivation in cones. (**a,b)** Retinal morphology analyzed at 4, 12 and 26 weeks of age in *RD*^*ΔHif1a*^ **(a)** and *AC*^*ΔHif1a*^ **(b)** mice and their respective Cre-negative controls (*RD*^*ctrl*^ and *AC*^*ctrl*^). (**c,d)** Immunostaining for OPN1SW in retinal cross-sections of the central and peripheral retina of 4- and 26-week-old *RD*^*ΔHif1a*^ **(c)** and *AC*^*ΔHif1a*^ **(d)** mice and their respective controls (*ctrl*). Cell nuclei were counterstained with DAPI. (**e,f)** Blood vessels labelled with isolectin on retinal flat mounts of *RD*^*ΔHif1a*^ **(e)** and *AC*^*ΔHif1a*^ **(f)** mice and their respective controls at 4 weeks of age. Vessels are highlighted with pseudocoloring of the 3 vascular plexi (deep (D): green; intermediate (I): red; primary (P): blue). Lower panels show the intermediate plexus separately. Scale bars as indicated.

To determine a potential effect of the cone-specific deletion of *Hif1a* transcription factor on gene expression in normal oxygen conditions, we measured mRNA levels of several genes during ageing (up to 26-weeks of age) (Fig. 2). First, we analyzed genes known to be HIF1 targets, such as vascular endothelial growth factor (*Vegf*), egl-9 family hypoxia inducible factor 1 (*Egln1*) and adrenomedullin (*Adm*). Even though mice were kept in normoxic conditions, *Vegf* and *Egln1* levels were reduced at the 4 weeks time point in *AC*^*ΔHif1a*^ but not in *RD*^*ΔHif1a*^ mice. Whether this reflects basic HIF1-activity in normoxic cones of *AC* mice at this time point needs further investigation. No compensatory increase in *Hif2a* (*Epas1*) expression was detected in *RD*^*ΔHif1a*^ and *AC*^*ΔHif1a*^ mice (Fig. 2a). Similar to *Hif1a* (Fig. 1h), 12-week-old *AC*^*ΔHif1a*^ mice had even reduced basal retinal expression of *Hif2a* (Fig. 2a). Levels of the cone-specific transcripts *Opn1sw* and *Opn1mw* were not or only marginally affected. Importantly, no increase but rather a decrease in *Casp1* transcription levels was observed in *RD*^*ΔHif1a*^ and *AC*^*ΔHif1a*^ mice, and no differential expression of the inflammatory markers glial fibrillary acidic protein (*Gfap*) and chemokine (C-C motif) ligand 2 (*Ccl2*) was apparent (Fig. 2b). This indicates that inactivation of *Hif1a* in cones of the mouse retina did not lead to degenerative or inflammatory processes. Indeed, analysis of retinal morphology and S-opsin localization revealed no signs of degenerative processes in *RD*^*ΔHif1a*^ mice up to 26 weeks of age (Fig. 3a,c). Although the retina of AC mice slowly degenerates over time^24^, no enhanced degeneration, reduced cone opsin expression or altered opsin localization was observed in *AC*^*ΔHif1a*^ mice when compared to age-matched controls (Fig. 3b,d). Furthermore, no indication of degenerative processes was noted in the inner retinas of either mouse line (Fig. 3a,b and data not shown).

Since an early inactivation of HIF1A in most cells of the peripheral retina prevents the formation of the intermediate vascular plexus^27^, we analyzed the retinal vasculature in 4-week-old *RD*^*ΔHif1a*^ and *AC*^*ΔHif1a*^ mice. Immunofluorescence stainings for isolectin B4 showed that *Hif1a* ablation in cones had no impact on formation of the three vascular plexi, both in the retinal center and periphery (Fig. 3e,f). Since BP-Cre is active as early as postnatal day 1 (data not shown), at a time before the formation of the vascular plexi, this indicates that *Hif1a* is needed in other cells than cones for proper vascularization during retinal postnatal development.

**Figure 4:**
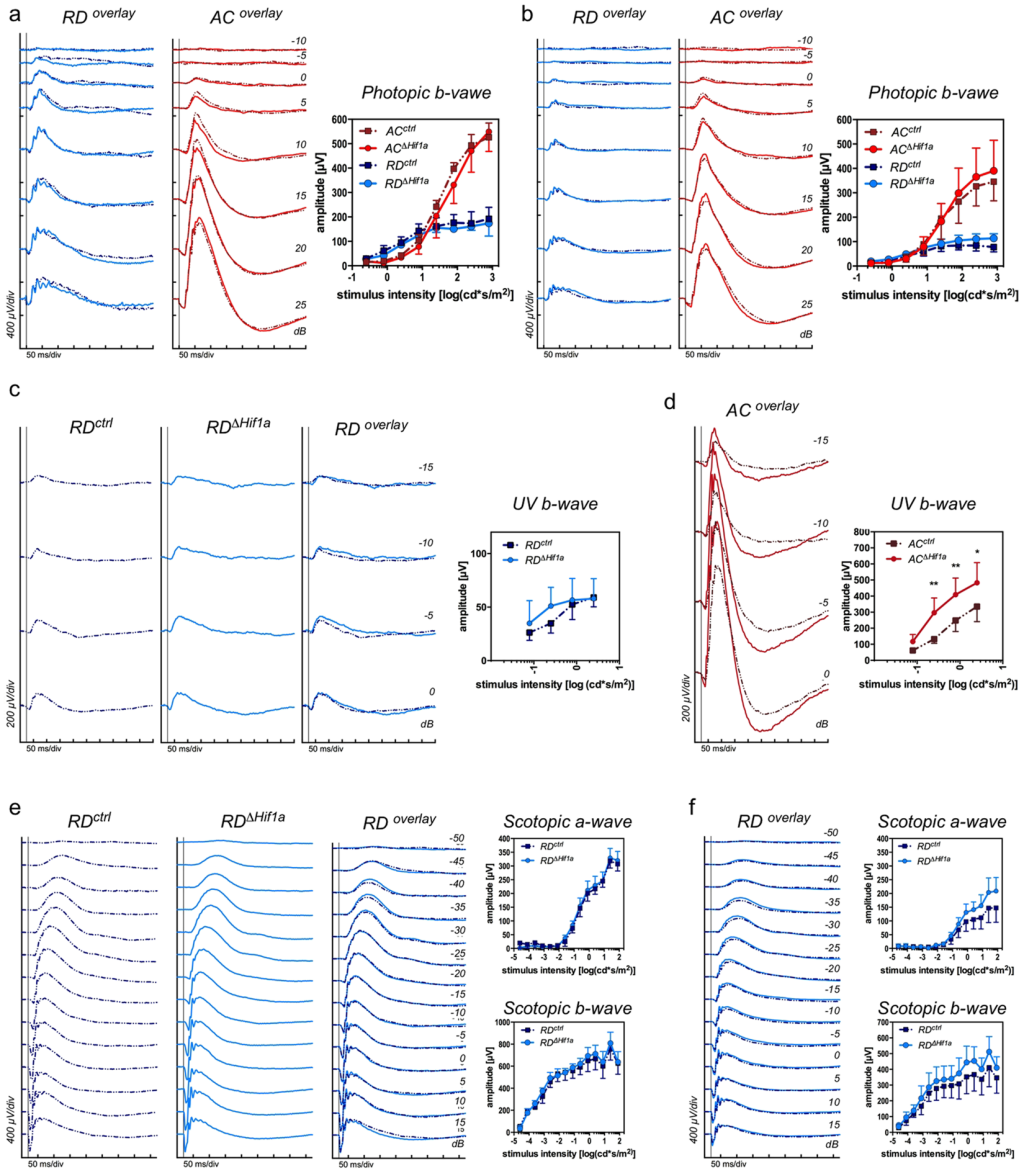
Retinal function in *RD*^*∆Hif1a*^ and *AC*^*∆Hif1a*^ mice. Averaged light-adapted **(a–d)** and dark-adapted **(e,f)** single flash ERG traces of *RD*^*ΔHif1a*^ and *AC*^*ΔHif1a*^ mice and their respective control littermates. A- and b-wave amplitudes were plotted as a function of light intensity (right panels, a–f). Photopic **(a,b)** and scotopic **(e,f)** single white-light flash intensity series at 12- **(a,e)** and 26- **(b,f)** weeks of age. Photopic single UV-light flash intensity series in 3-month-old *RD*^*ΔHif1a*^ **(c)** and *AC*^*ΔHif1a*^ **(d)** mice and their respective control littermates. Shown are means ± SD of n ≥ 3 (*ctrl*/kd = **a** (RD 5/4; AC 4/3); **b** (RD 4/4; AC 4/4); **c** (6/6); **d** (3/3); **e** (4/4); **f** (4/4)). Two-way ANOVA with Šídák’s multiple comparison test was used for statistical analysis. *p < 0.05; **p < 0.01.

To further confirm that *Hif1a* deletion did not lead to cone degenerative processes and/or altered functionality we analyzed retinal function by electroretinography (ERG) at 12 weeks and 6 months of age. Data shows that cone-specific deletion of *Hif1a* did not affect light-adapted ERG responses to flashes of white light in RD or AC mice (Fig. 4a,b). Wave forms and wave amplitudes did not differ between *RD*^*ctrl*^ and *RD*^*ΔHif1a*^ or between *AC*^*ctrl*^ and *AC*^*ΔHif1a*^ mice at both time points (Fig. 4a,b). Furthermore, light-adapted ERG elicited with UV light (365 nm), to which mouse S-opsin pigments are most sensitive^28^, did not lead to differences in b-wave amplitudes between *RD*^*ctrl*^ and *RD*^*ΔHif1a*^ mice (Fig. 4c). Interestingly, *AC*^*ΔHif1a*^ mice exhibited increased b-wave amplitudes when stimulated with UV light (Fig. 4d), an unexpected finding requiring further exploration. As expected, scotopic function of *RD*^*ΔHif1a*^ mice was similar to their controls at 12 weeks and 6 months of age (Fig. 4e,f). This suggests that lack of *Hif1a* in cones did not negatively affect retinal function up to 6 months of age.

**Figure 5:**
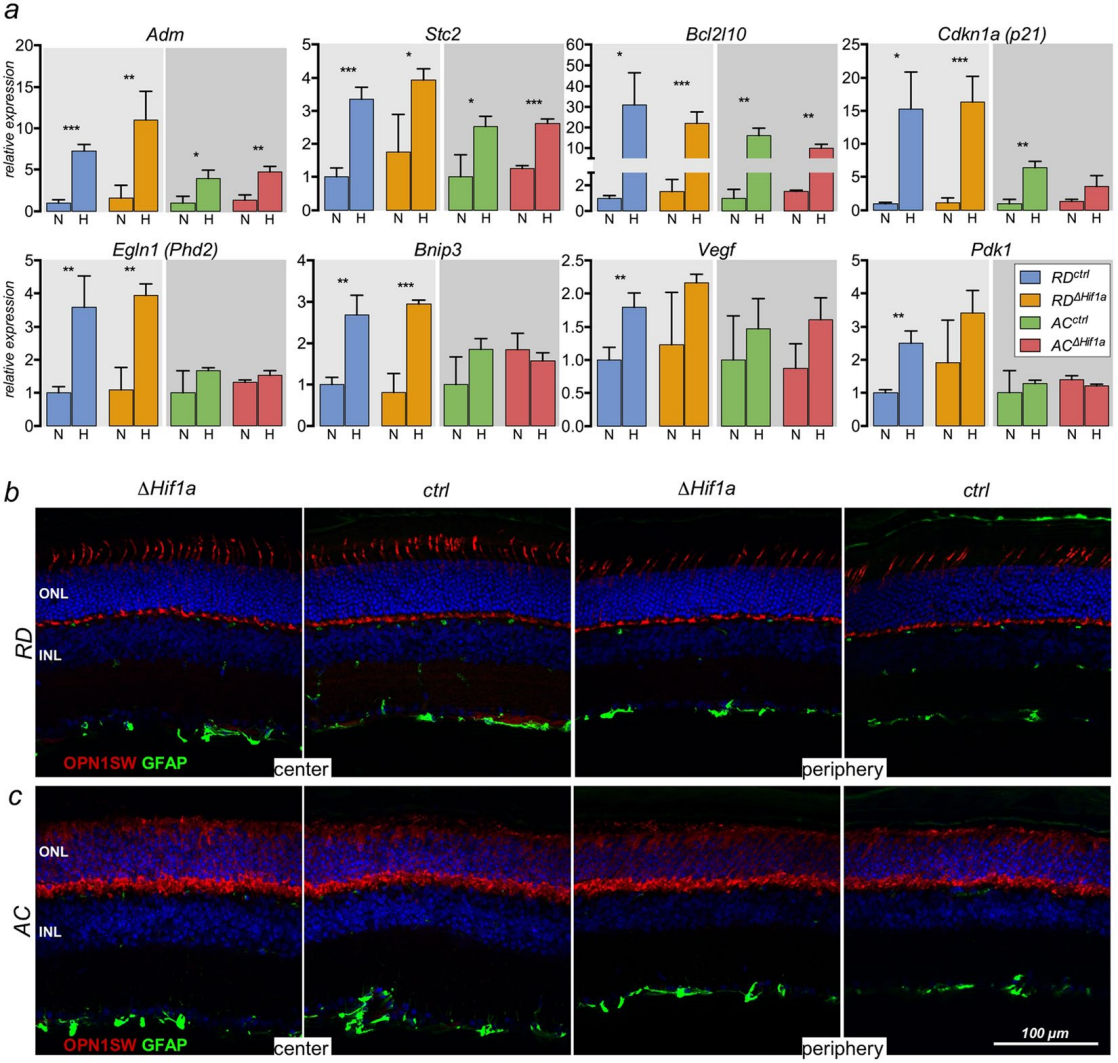
Retinal hypoxia tolerance in the *RD*^*∆Hif1a*^ and *AC*^*∆Hif1a*^ mice. (**a)** *RD*^*ΔHif1a*^, *RD*^*ctrl*^, *AC*^*ΔHif1a*^ and *AC*^*ctrl*^ mice were kept under normoxic conditions (N) or exposed to hypoxia (H, 7% O_2_, 6 h). Gene expression levels in total retinal RNA were determined immediately after hypoxic exposure. Expression levels were normalized to *Actb* and shown relative to the control littermates under normoxic conditions, which levels were set to 1 for each group (RD and AC). Shown are means ± standard deviation (SD) for n = 3. The differences in gene expression levels between normoxic and hypoxic conditions were tested for significance using a two-way ANOVA with Šídák’s multiple comparison test *p < 0.05; **p < 0.01; ***p < 0.001. (**b)** Detection of OPN1SW (red) and GFAP (green) in sections of the central or peripheral retina of *RD*^*ΔHif1a*^, *AC*^*ΔHif1a*^ and their respective controls at 10 days after hypoxic exposure. Cell nuclei were counterstained with DAPI (blue). ONL: outer nuclear layer, INL: inner nuclear layer. All mice were 12-weeks of age. Scale bar as indicated.

To test the potential influence of the cone-specific inactivation of *Hif1a* on the general hypoxic response in retinas of *RD*^*ΔHif1a*^ and *AC*^*ΔHif1a*^ mice, we analyzed retinal gene expression after exposure of mice to 7% oxygen for 6 hours (Fig. 5a). Hypoxic exposure increased expression of several hypoxia-responsive genes including *Adm*, *Stc2* (stanniocalcin 2), *Bcl2l10* (Bcl2 like 10) *Cdkn1a* (cyclin dependent kinase inhibitor 1A, alias p21), *Egln1*, *Bnip3* (bcl2 interacting protein 3), *Vegf* and *Pdk1* in *RD*^*ctrl*^ mice (Fig. 5a). All these genes were also upregulated by hypoxia in *RD*^*ΔHif1a*^ mice that lack *Hif1a* in cones, even though *Vegf* and *Pdk1* did no longer reach significance (Fig. 5a). Likely, the low number of retinal cells without functional HIF1A did not allow to detect possible differences in expression levels. Alternatively, cones did not or not strongly express these genes. The hypoxic response in *AC*^*ctrl*^ mice was less pronounced with only *Adm*, *Stc2*, *Bcl2l10* and *Cdkn1a* reaching significance. Even though *Cre* expression and *Hif1a* deletion was widespread in cones of the AC retina (Fig. 1), only *Cdkn1a* was less strongly induced by hypoxia while hypoxic induction of *Adm*, *Stc2* and *Bcl2l10* in *AC*^*ΔHif1a*^ mice was similar to their controls. This may indicate that cones react less strongly to acute hypoxia in general and/or that these genes are not strong HIF1 targets in cones of the AC mouse. Indeed, the expression of *Phd2*, *Bnip3, Vegf* and *Pdk1* in hypoxic AC retinas was not increased, which may support the above hypothesis. Alternatively, these genes may mainly be regulated in rods, the cells that are absent in AC mice. Clearly, this is an interesting point that needs further clarification by analyzing the response of cones to hypoxia in greater detail.

Despite the attenuated response of cones to acute hypoxia, the HIF1A transcription factor may nevertheless be important for the cellular adaptation of cones to reduced oxygen levels. To test whether cones require functional HIF1A to survive reduced oxygen conditions, we analyzed the distribution of cones 10 days following acute hypoxia. No signs of gliosis or changes in cone density were observed in the *RD*^*ΔHif1a*^, the *AC*^*ΔHif1a*^ or their respective control mice as revealed by GFAP and OPN1SW immunofluorescence (Fig. 5b,c).

**Figure 6:**
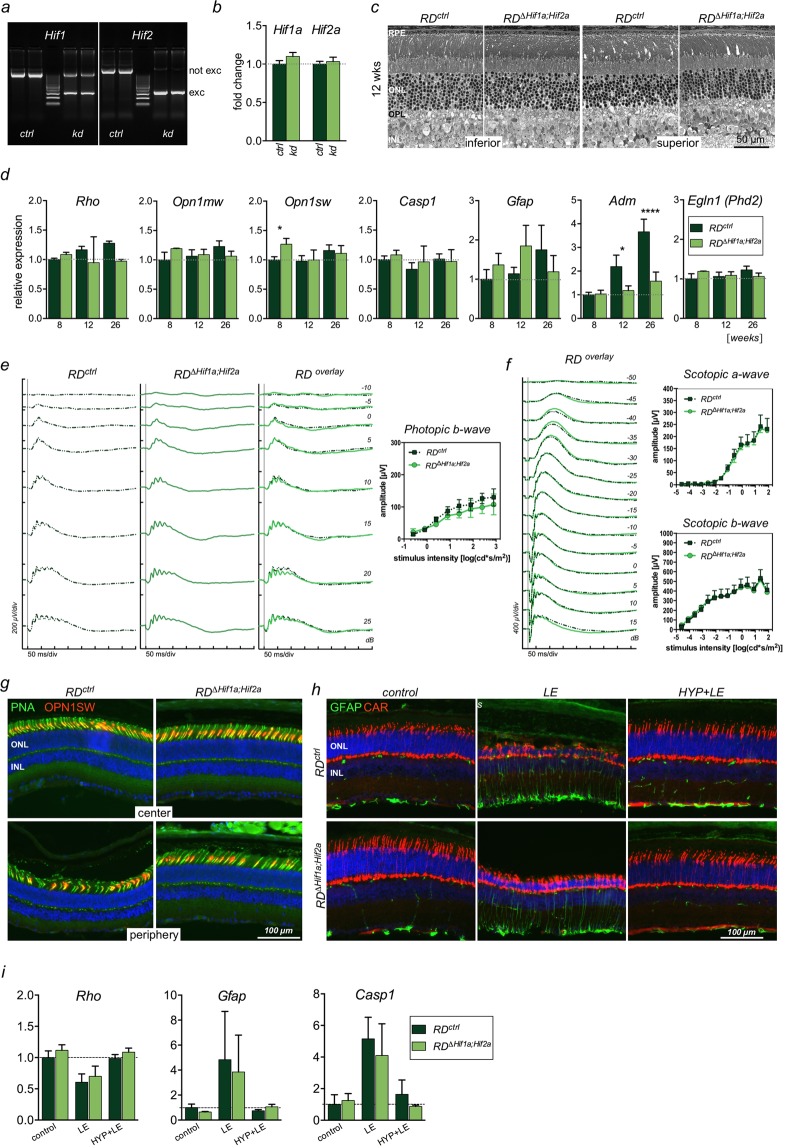
Cones are not affected in *RD*^*ΔHif1a;Hif2a*^ mice. **(a)** Detection of *Hif1a* (left) and *Hif2a* (right) deletion fragment (exc) in genomic DNA isolated from retinas of in *RD*^*ΔHif1a;Hif2a*^ mice (kd) and their control littermates (ctrl). Not exc: not excised, floxed DNA sequence. (**b)** Relative expression levels of *Hif1a* and *Hif2a* mRNA in retinas of 8-week-old control (ctrl) and *RD*^*∆Hif1a;Hif2a*^ (kd) mice determined by semi-quantitative real-time PCR. (**c)** Retinal morphology of *RD*^*ΔHif1a;ΔHif2a*^ mice and their respective Cre-negative controls (*RD*^*ctrl*^) at 12 weeks of age. RPE, retinal pigment epithelium; ONL, outer nuclear layer; INL, inner nuclear layer. (**d)** Relative levels of indicated mRNAs in retinas of 8, 12 and 26-week-old *RD*^*∆Hif1a;Hif2a*^ mice and their *RD*^*ctrl*^ littermates determined by semi-quantitative real-time PCR. (**e,f)** ERG recordings in 3-month-old *RD*^*∆Hif1a;Hif2a*^ mice and their control littermates. Averaged traces evoked by single flashes of white light with increasing intensities under light-adapted photopic **(e)** and dark-adapted scotopic **(f)** conditions. The a- and b-wave amplitudes (right panels) were plotted as a function of light intensity and shown as means ± SD of n = 3. (**g)** Cones detected with peanut agglutinin (PNA, green) and OPN1SW (red) staining in central or peripheral retinal sections of 12-week-old *RD*^*ΔHif1a;Hif2a*^ and their respective control (*RD*^*ctrl*^) mice. (**h,i)** Light damage susceptibility of *RD*^*∆Hif1a;Hif2a*^ and *RD*^*ctrl*^ mice after hypoxic preconditioning. Mice were exposed to high levels of white light without (middle panels; LE) or were hypoxia-preconditioned before (right panels; HYP + LE) and analyzed 10 days following the insult by immunofluorescence (**h**) or semi-quantitative real-time PCR (**i**). Control animals were not subjected to any treatment (control). By the time of death mice were 12- to 14-week-old. Cone arrestin (CAR, red) and GFAP (green) immunoreactivity was analyzed on retinal cross-sections. Cell nuclei were counterstained with DAPI (blue). ONL: outer nuclear layer, INL: inner nuclear layer. Scale bars as indicated. mRNA levels (**b,d,i**) were normalized to *Actb* and expressed relatively to *ctrl*, which were set to 1. Shown are means ± SD of n = 3. Two-way ANOVA with Šídák’s multiple comparison test was used for all statistical analyses.

Since lack of *Hif1a* in cones did not reveal any signs of impaired survival or function, we asked whether *Hif2a* might compensate for *Hif1a*. Therefore, we tested *RD*^*∆Hif1a;Hif2a*^ mice which lacked both transcription factors in S-cones. PCR amplification of *Hif1a* and *Hif2a* sequences from retinal genomic DNA showed expected excision fragments, indicating successful inactivation of both genes in cones upon *Cre* expression (Fig. 6a). Deletion of *Hif1a* and *Hif2a* floxed sequences in S-cones of the normal retina had no measurable impact on general *Hif1a* and *Hif2a* transcript levels determined in total retinal RNA (Fig. 6b). Retinal morphology of 12-week-old mice was indistinguishable between *RD*^*∆Hif1a;Hif2a*^ and *RD*^*ctrl*^ mice (Fig. 6c). The levels of rod- (*Rho*) and cone-specific (*Opn1mw, Opn1sw*) transcripts were similar in mice up to 6 months of age, apart from transiently increased expression of *Opn1sw* in *RD*^*∆Hif1a;Hif2a*^ mice at 8 weeks (Fig. 6d). Accordingly, no signs of increased expression of degeneration- (*Casp1*) or gliosis-associated (*Gfap*) genes were observed in *RD*^*∆Hif1a;Hif2a*^ mice. Interestingly, as opposed to increasing expression levels of *Adm* in controls, steady state levels of *Adm* were observed during ageing in *RD*^*∆Hif1a;Hif2a*^ mice. Expression of another HIF-regulated gene *Egln1* remained constant in both mouse lines during ageing (Fig. 6d).

Light-adapted ERG showed no significant differences in waveforms and b-wave amplitudes in *RD*^*∆Hif1a;Hif2a*^ mice in response to white light stimuli (Fig. 6e). Likewise, the dark-adapted scotopic function was not affected (Fig. 6f). Furthermore, mice lacking HIF1A and HIF2A showed no apparent cone loss and a similar cone distribution in central and peripheral retinas was noticed in control and *RD*^*∆Hif1a;Hif2a*^ mice (Fig. 6g). Collectively, these data suggested that the combined absence of *Hif1a* and *Hif2a* had no impact on cone function and cone survival under normal physiological conditions.

To test whether functional HIF1A and HIF2A are required in cones under pathological conditions we used the paradigm of neuroprotection by hypoxic preconditioning, in which short-term hypoxia prior to exposure of mice to toxic light levels protects rod photoreceptors against light-induced retinal degeneration^29,30^. *RD*^*∆Hif1a;Hif2a*^ mice and their respective controls (*RD*^*ctrl*^) were exposed to light with or without hypoxic preconditioning and analyzed 10 days following the insult (Fig. 6h). Based on GFAP immunoreactivity, cone arrestin distribution and ONL thickness, retinas of both mouse lines were similarly susceptible to light-induced degeneration (Fig. 6h, LE) and were similarly protected from damage by hypoxic preconditioning (Fig. 6h, HYP + LE). Furthermore, we have analyzed gene expression of rhodopsin (*Rho)*, *Gfap* and *Casp1* (Fig. 6i) in mice subjected to the same experimental setup. A similar, statistically not different pattern of expression was observed for all three genes in *RD*^*∆Hif1a;Hif2a*^ and *RD*^*ctrl*^ mice. Upon light exposure *Rho* levels were reduced, likely due to the loss of photoreceptors, while *Gfap* and *Casp1* were similarly increased in the two mouse lines 10 days following the light insult (Fig. 6i, LE). Hypoxic preconditioning conferred similar protection against light-induced degeneration in both mouse lines (Fig. 6i, HYP + LE). These data show that cones do not require functional HIF1A and HIF2A to survive under normal or pathological conditions and that hypoxia-mediated rod protection is not facilitated *in trans* by HIF1A and/or HIF2A activation in cones.

## Discussion

Evidence suggests that the ageing retina experiences a mild but chronic hypoxic insult that potentially contributes to the development of age-related diseases such as AMD^5,6^. It has been shown that chronic activity of the major hypoxia-responsive transcription factors HIF1 in photoreceptors^12,31^ and HIF2 in the RPE^11^ leads to retinal degeneration and vision loss. Reducing HIF levels may therefore be a valid approach to prevent or slow disease progression in the ageing eye. However, as HIF1 and HIF2 regulate expression of a large number of genes and metabolic pathways involved in angiogenesis, glycolysis, ischemia, cell survival, proliferation and others^32,33^, it is important to evaluate the safety and potential adverse effects of such an approach.

It was already shown that inactivation of *Hif1a* and *Hif2a* in the adult RPE^34,35^ or rod photoreceptors^22,23^ is well tolerated and without a noticeable loss in cell or tissue integrity, or a decline of retinal function. In this study, we examined the consequences of a cone-specific *Hif1a* single or a *Hif1a* and *Hif2a* double knockout for retinal physiology. Our data indicate that absence of functional HIF1A in adult cones did not affect retinal morphology, function or photoreceptor survival, even after a hypoxic challenge. Lack of HIF1A (together with HIF2A) in cones also did not affect protection against light-induced retinal degeneration by hypoxic preconditioning. This suggests that HIF-driven neuroprotective factors released by cones are not responsible for hypoxic protection of rods following exposure to toxic light levels.

Regulation of genes by HIF transcription factors has been studied for decades and several genes have been identified as targets for HIF1, HIF2 or both transcription factors^36^. Under some conditions, however, a specific isoform can substitute for the lack of the other and drive expression of the respective genes^33^. In addition, depending on cell type and tissue context, an interplay between HIF1 and HIF2 may fine-tune various processes relevant to cellular metabolism^37^. Although glycolysis is a major pathway regulated mainly by HIF1-controlled expression of glycolytic enzymes^36,38^, HIF2 may also be involved and have a modulatory function, for example by contributing to the regulation of *Glut1* (*Slc2a1*), a main glucose transporter important for cones^39^. Since cones produce cellular energy also through aerobic glycolysis^39^, efficient glucose uptake and proper glycolytic function are essential for cone metabolism. Because cones do not saturate and consume more ATP than rods in bright light^40^, even subtle metabolic changes potentially inflicted by the absence of HIF transcription factors might therefore be expected to affect cone function. This may not only be assumed for hypoxic but also for normoxic conditions since HIF1 may also be partially active in non-hypoxic cells^41^, and basic HIF1A levels were demonstrated in retinas of both RD^31^ and AC^12^ mice. It may thus be somewhat surprising that lack of *Hif1a* or of *Hif1a* and *Hif2a* in adult cones was without obvious phenotype. The lack of an apparent role of HIF1 in adult photoreceptors and other cells including hematopoietic stem cells^42^ contrasts the importance of HIF1 in various cellular systems including myeloid cell mediated inflammation^43^, energy metabolism and calcium flux in the normoxic heart^44^, chondrogenesis^45^ and others^46^. It also contrasts the significant function of HIF1 during retinal development where tissue-specific inactivation prevents formation of the intermediate vascular plexus of the retina^27^. The central role of HIF1 during general development is further underlined by the embryonic lethality of systemic *Hif1a* knockout mice due to vascular abnormalities^47^.

Importantly, however, the lack of a retinal phenotype in *RD*^*∆Hif1a*^ and *AC*^*∆Hif1a*^ mice indicates that adult cones in the mature retina survive and function normally in the absence of HIF1A, as rods do^22,23^. This may indicate that an anti-HIF strategy for the treatment of retinal degenerative diseases involving hypoxic component in the adult or ageing retina might be feasible and safe. Such a strategy is currently being developed in our lab and will target HIF1A in photoreceptors and HIF2A in the RPE (see above) by an RNA interference approach using AAV-mediated gene therapy.

### Research design and methods

#### Mice

Animal maintenance and experimentation adhered to the regulations of the veterinary authorities of Kanton Zurich, Switzerland and the Statement for the Use of Animals in Ophthalmic and Vision Research. The protocol was approved by the veterinary authorities of Kanton Zurich (license nr. 141/2016). Mice were housed in the animal facility of the University of Zurich and maintained in a 14 hours light: 10 hours dark cycle with access to food and water *ad libitum*. Single mutant *Rpe65*^*R91W*^ (*Rpe65*^*tm1Lrcb*^)^48^ and *Nrl*^*−/−*^ (*Nrl*^*tm1Asw*^)^26^ mice were used to generate the double mutant *Rpe65*^*R91W*^*;Nrl*^*−/−*^ (*R91W;Nrl*^*−/−*^) all-cone (AC) mouse as previously described^24^. To test for the expression of the Cre recombinase in the normal, rod-dominant (RD) mouse retina, *BPCre* mice (*Tg(Opn1sw-cre)1Asw*)^25^ were bred to a *ZsGreen* reporter line (*Ai6 mice, Gt(ROSA)26Sor*^*tm6(CAGZsGreen1) Hze*^)^49^. The expression of Cre recombinase in all-cone retinas of *R91W/Nrl*^*−/−*^ mice was analyzed as described recently^12^.

To delete *Hif1a* and *Hif2a* (*Epas1*) from cones in RD retinas, the *Hif1a*^*f/f*^ (*Hif1a*^*tm3Rsjo*^)^50^ and *Hif2a*^*f/f*^ (*Epas1*^*tm1Mcs*^)^51^ mouse strains were crossed with *BPCre* mice to generate either *BPCre;Hif1a*^*f/f*^ (=*RD*^*ΔHif1a*^) or *BPCre;Hif1a*^*f/f*^*;Hif2a*^*f/f*^ (=*RD*^*ΔHif1a;Hif2a*^). To delete *Hif1a* from cones in AC retinas, the *Hif1a*^*f/f*^ mice were mated to *R91W;Nrl*^*−/−*^ and *BPCre* mice, which resulted in the generation of the quadruple *BPCre*;*R91W;Nrl*^*−/−*^*;Hif1a*^*f/f*^ (=*AC*^*ΔHif1a*^) mouse line. Cre-negative littermates served as controls: *Hif1a*^*f/f*^ and *Hif1a*^*f/f*^*;Hif2a*^*f/f*^ (=*RD*^*ctrl*^) and *R91W;Nrl*^*−/−*^*;Hif1a*^*f/f*^ (=*AC*^*ctrl*^), respectively.

Mice were genotyped by PCR amplification of genomic DNA from ear biopsies by specific primer pairs (Table 1). Deletion of *Hif1a* and *Hif2a* floxed sequences was confirmed by PCR using genomic DNA isolated from retinal tissue and appropriate primer pairs (Table 1).

**Table 1:** Primer sequences used for genotyping of mouse lines and detection of Cre-recombined alleles.

Gene		primer	product (bp)
*BPCre*	forward	GGACATGTTCAGGGATCGCCAGGCG	268
	reverse	GCATAACCAGTGAAACAGCATTGCTG	
*Nrl (ko)*	forward	TGAATACAGGGACGACACCA	400
	reverse	GTTCTAATTCCATCAGAAGCTGAC	
*Nrl (wt)*	forward	GTG TTC CTT GGCTGGAAAGA	250
	reverse	CTGTTCACTGTGGGCTTTCA	
*Hif1a (f/f)*	forward	GCAGTTAAGAGCACTAGTTG	260 floxed
	reverse	GGAGCTATCTCTCTAGACC	215 wt
*ΔHif1a*	forward	TTGGGGATGAAAACATCTGC	270 excised
	reverse	GCAGTTAAGAGCACTAGTTG	
*ΔHif1a*	forward	GGAGCTATCTCTCTAGACC	260 floxed
	reverse	GCAGTTAAGAGCACTAGTTG	215 wt
*Hif2a (f/f)*	forward	GAGAGCAGCTTCTCCTGGAA	220 floxed
	reverse	TGTAGGCAAGGAAACCAAGG	182 wt
*ΔHif2a*	forward	GCTAACACTGTACTGTCTGAAAGAGTAGC	>1000 floxed
	reverse	GAGAGCAGCTTCTCCTGGAA	app 320 wt
*ZsGreen (wt)*	forward	AAGGGAGCTGCAGTGGAGTA	279
	reverse	CCGAAAATCTGTGGGAAGTC	
*ZsGreen (tg)*	forward	AACCAGAAGTGGCACCTGAC	199
	reverse	GGCATTAAAGCAGCGTATCC	
*Rpe65(R91W)*	forward	GCTGGTCTTGCCTGTATCA	998^a^
	reverse	GTCAGAGACAGTGCTGTGTT	

^a^PCR product digested by TaqI gives fragments of 619 + 379 (wt) or remains uncut (R91W).

#### RNA isolation, reverse transcription and real-time PCR

Retinas collected at different ages were isolated through a slit in the cornea, frozen in liquid nitrogen and stored at −80 °C. Total RNA was isolated using an RNA isolation kit (RNeasy, Qiagen, Hilden, Germany or Nucleo Spin RNA, Macherey Nagel, Oensingen, Switzerland) according to manufacturer’s instructions with an additional on-column DNaseI treatment. First-strand cDNA synthesis was catalysed by M-MLV reverse transcriptase (Promega, Dübendorf, Switzerland) using oligo(dT) primers and 1 μg of RNA. Gene expression was analyzed by semiquantitative real-time PCR (QuantStudio 3, ThermoFisher Scientific, Bremen, Germany) using 10 ng of cDNA template and PowerUp SYBR Master Mix (ThermoFisher Scientific). Primer pairs (Table 2) were designed to span large intronic regions and to avoid single nucleotide polymorphisms. Reactions were normalized to β-Actin (*Actb*) and relative expression was calculated by the comparative threshold cycle method (ΔΔC_T_). Three mice per group were analyzed. All data are presented as mean values ± standard deviation (SD). See bellow for statistical analysis.

**Table 2:** Primer sequences used for semi quantitative real-time PCR analyses.

Gene		primer	product (bp)
*Actb*	forward	CAACGGCTCCGGCATGTGC	153
	reverse	CTCTTGCTCTGGGCCTCG	
*Cre* ^53^	forward	TAAACTGGTCGAGCGATGGA	187
	reverse	ACCAGAGTCATCCTTAGCGC	
*Hif1a*	forward	TCATCAGTTGCCACTTCCCCA	198
	reverse	CCGTCATCTGTTAGCACCATC	
*Vegfa*	forward	ACTTGTGTTGGGAGGAGGATGTC	171
	reverse	AATGGGTTTGTCGTGTTTCTGG	
*Egln1*	forward	GCAGCATGGACGACCTGAT	123
	reverse	CAACGTGACGGACATAGCCT	
*Adm*	forward	TCCTGGTTTCTCGGCTTCTC	133
	reverse	ATTCTGTGGCGATGCTCTGA	
*Epas1*	forward	GGAGCTCAAAAGGTGTCAGG	61
	reverse	CAGGTAAGGCTCGAACGATG	
*Opn1sw*	forward	TGTACATGGTCAACAATCGGA	153
	reverse	ACACCATCTCCAGAATGCAAG	
*Opn1mw*	forward	CTCTGCTACCTCCAAGTGTGG	154
	reverse	AAGTATAGGGTCCCCAGCAGA	
*Casp1*	forward	GGCAGGAATTCTGGAGCTTCAA	138
	reverse	GTCAGTCCTGGAAATGTGCC	
*Gfap*	forward	CCACCAAACTGGCTGATGTCTAC	240
	reverse	TTCTCTCCAAATCCACACGAGC	
*Ccl2*	forward	GGCTCAGCCAGATGCAGTTA	108
	reverse	CTGCTGCTGGTGATCCTCTT	
*Stc2*	forward	AGCAGGAAGTGTCCAGCAAT	166
	reverse	GGTTCACAAGGTCCACATAGG	
*Bcl2l10*	forward	GAACTTTCTGTATAATCTGCTCATGG	89
	reverse	TGAAGAAGCGGCAAAAGC	
*Cdkn1a*	forward	CGGTGTCAGAGTCTAGGGGAATTG	238
	reverse	CGTGACGAAGTCAAAGTTCCACC	
*Bnip3*	forward	CCTGTCGCAGTTGGGTTC	93
	reverse	GAAGTGCAGTTCTACCCAGGAG	
*Pdk1*	forward	GTTGAAACGTCCCGTGCT	170
	reverse	AGTCTCTCGACGGATTCTGT	
*Rho*	forward	CTTCACCTGGATCATGGCGTT	130
	reverse	TTCGTTGTTGACCTCAGGCTTG	

#### Morphology and immunofluorescence

Mice were euthanized, their eyes marked nasally and enucleated. For analysis of retinal morphology eyes were fixed in 2.5% glutaraldehyde in cacodylate buffer (pH 7.2, 0.1 M), as described previously^52^. Nasal and temporal eyecup halves were embedded in epon plastic. Semi-thin cross sections (0.5 μm) were counterstained with toluidine blue and analyzed by light microscopy (Axioplan, Zeiss, Jena, Germany). Representative images were created by merging 2 micrographs acquired at 40x using Photoshop CS5 photomerge tool (Adobe Systems, Inc., San Jose, CA, USA).

For immunofluorescence, the eyes were fixed in 4% paraformaldehyde (PFA) in phosphate buffer (PBS) as described recently^12^. Following lens removal and post-fixation, the eyecups were cryoprotected in sucrose, embedded, frozen and stored at −80 °C until sectioning. Cryosections (12 μm) were blocked with 3% normal goat serum (Sigma-Aldrich, St. Louis, MO, USA) or 10% horse serum (Sigma) in PBS containing 0.3% Triton X-100 (Sigma). The sections were incubated overnight at 4 °C with the following primary antibodies: rabbit anti-OPN1MW (1:500, AB5405; Merck, Darmstadt, Germany), goat anti-OPN1SW (1:500, sc-14363; Santa Cruz Biotechnology, Santa Cruz, US), mouse anti-GFAP (1:250, G3893-Clone GA-5, Sigma, Buchs, Switzerland) and rabbit anti-CAR (cone arrestin, 1:1000, AB15282, Merck). Fluorescein isothiocyanate (FITC)-conjugated peanut agglutinin (PNA, 1:250, L7381, Sigma) was also used to stain cones. Fluorescence signal was analyzed using a fluorescence microscope (Axioplan, Zeiss). On average 8 images, acquired at 10x magnification, were combined to reconstitute retinal panoramas using Photoshop photomerge tool (Adobe Systems). Images shown are representative of n ≥3 mice per genotype and age group.

#### Retinal whole mounts and visualization of the retinal vasculature

Eyes from 4-week-old mice were isolated and fixed in 2% PFA (in PBS) for 5 to 10 minutes, as described recently^27^. Cornea and lens were removed, the retina dissected, flatmounted in PBS and post-fixed in 4% PFA for 1 h at room temperature. After blocking (3% normal goat serum, 0.3% Triton X-100 in PBS, 1 h) whole mounts were incubated with isolectin GS-IB4-Alexa594 (1:300, I21413; Thermo Fisher Scientific, Waltham, MA, USA) at 4 °C overnight. Whole mounts were washed in PBS, flattened, mounted on glass slides and analyzed by fluorescence microscopy (Axioplan/ApoTome; Zeiss). Images were taken at focal points that were 0.4 µm apart (Z-stacking) using a 10x objective. On average, 80 Z-stack images were acquired from central and 60 from peripheral retinal regions. Representative images for each of the three vascular plexi were selected, pseudocolored and superimposed using Photoshop CS5 (Adobe Systems). Shown are representative images of n = 3 mice per genotype.

#### Acute hypoxia and light damage

Mice were placed in a hypoxic chamber and exposed to hypoxic conditions as previously described^23^. Briefly, the oxygen concentration was decreased to 7% by gradually altering the O_2_:N_2_ ratio over a period of 1 h. After 6 h at 7% O_2_ (hypoxia), mice were immediately euthanized or kept in normal room air for additional 10 days. Control groups not subjected to hypoxia were processed in parallel. A subset of mice exposed to hypoxia was subjected to light damage after a 4 h period of dark-adaptation in normal room air. Mice with dilated pupils (1% cyclogyl (Alcon, Cham, Switzerland) and 5% phenylephrine (Ciba Vision, Niederwangen, Switzerland)) were then exposed to 13′000 lux of white fluorescent light for 2 h and analyzed 10 days afterwards. Control groups were exposed only to light or were not subjected to any treatment. Ocular tissue of mice between 12 and 14 weeks of age was processed as described in the RNA isolation and immunofluorescence sections.

#### Electroretinography (ERG)

Mice were processed for ERG as described^23^. Briefly, after dark-adaptation overnight and pupil dilation, mice were anesthetized by a subcutaneous injection of a mixture of ketamine (85 mg/kg, Parke-Davis, Berlin, Germany) and rompun (xylazine (4 mg/kg, Bayer AG, Leverkusen, Germany)) and their eyes lubricated. Electroretinograms were recorded simultaneously from both eyes using a UTAS BigShot Ganzfeld light source (LKC Technologies, Inc. Gaithersburg, MD, USA). Fourteen flash intensities ranging from −50 dB (−4.6 log[cd * s/m^2^]) to 15 dB (1.9 log[cd * s/m^2^]) and eight flash intensities ranging from −10 dB (−0.6 log[cd * s/m^2^]) to 25 dB (2.9 log[cd * s/m^2^]) were used for dark- (scotopic) and light-adapted (photopic) single-flash intensity ERG series, respectively. The standard rod-suppressive background light (30 cd/m^2^) was used prior (5 min) and during recordings in photopic conditions. Light-adapted UV ERG (365 nm) was recorded with light intensities ranging from −15 dB (−1.1 log[cd * s/m^2^]) to 0 dB (0.4 log[cd * s/m^2^]). Ten recordings were averaged per light intensity. Traces obtained from both eyes were averaged for each light intensity. Averages of each mouse group (n ≥ 4) were then used to calculate the mean values and traces presented in figures. Significance was determined as described below.

#### Statistics

Statistical analysis was performed using Prism7 software (GraphPad, San Diego, CA, USA). All data are presented as mean values ± standard deviation (SD). The significance of data was determined by two-way ANOVA followed by Šídák’s multiple comparison test, except for Fig. 1 where one-way ANOVA and Tukey’s multiple comparison test was used. *p < 0.05; **p < 0.01; ***p < 0.001, ****p < 0.0001. n ≥ 3, as indicated in the figures.

## Data Availability

No datasets were generated or analyzed during the current study.
